# *Pseudomonas aeruginosa* glutathione biosynthesis genes play multiple roles in stress protection, bacterial virulence and biofilm formation

**DOI:** 10.1371/journal.pone.0205815

**Published:** 2018-10-16

**Authors:** Lampet Wongsaroj, Kritsakorn Saninjuk, Adisak Romsang, Jintana Duang-nkern, Wachareeporn Trinachartvanit, Paiboon Vattanaviboon, Skorn Mongkolsuk

**Affiliations:** 1 Molecular Medicine Graduate Program, Faculty of Science, Mahidol University, Bangkok, Thailand; 2 Department of Biotechnology, Faculty of Science, Mahidol University, Bangkok, Thailand; 3 Center for Emerging Bacterial Infections, Faculty of Science, Mahidol University, Bangkok, Thailand; 4 Laboratory of Biotechnology, Chulabhorn Research Institute, Bangkok, Thailand; 5 Department of Biology, Faculty of Science, Mahidol University, Bangkok, Thailand; 6 Program in Applied Biological Sciences: Environmental Health, Chulabhorn Graduate Institute, Bangkok, Thailand; East Carolina University Brody School of Medicine, UNITED STATES

## Abstract

*Pseudomonas aeruginosa* PAO1 contains *gshA* and *gshB* genes, which encode enzymes involved in glutathione (GSH) biosynthesis. Challenging *P*. *aeruginosa* with hydrogen peroxide, cumene hydroperoxide, and *t*-butyl hydroperoxide increased the expression of *gshA* and *gshB*. The physiological roles of these genes in *P*. *aeruginosa* oxidative stress, bacterial virulence, and biofilm formation were examined using *P*. *aeruginosa* Δ*gshA*, Δ*gshB*, and double Δ*gshA*Δ*gshB* mutant strains. These mutants exhibited significantly increased susceptibility to methyl viologen, thiol-depleting agent, and methylglyoxal compared to PAO1. Expression of functional *gshA*, *gshB* or exogenous supplementation with GSH complemented these phenotypes, which indicates that the observed mutant phenotypes arose from their inability to produce GSH. Virulence assays using a *Drosophila melanogaster* model revealed that the Δ*gshA*, Δ*gshB* and double Δ*gshA*Δ*gshB* mutants exhibited attenuated virulence phenotypes. An analysis of virulence factors, including pyocyanin, pyoverdine, and cell motility (swimming and twitching), showed that these levels were reduced in these *gsh* mutants compared to PAO1. In contrast, biofilm formation increased in mutants. These data indicate that the GSH product and the genes responsible for GSH synthesis play multiple crucial roles in oxidative stress protection, bacterial virulence and biofilm formation in *P*. *aeruginosa*.

## Introduction

*Pseudomonas aeruginosa* is an opportunistic human pathogen that causes nosocomial infections in hospitalized patients with AIDS, cancer, and cystic fibrosis (CF). During infection, *P*. *aeruginosa* is first eliminated by innate immune cells, such as phagocytic cells, in which NADPH oxidase-dependent reactive oxygen species (ROS) are generated as bactericidal substances [1]. ROS are also generated as a by-product of electron transport [2]. Oxidative stress occurs when cells are exposed to ROS, such as superoxide anion (O_2_·^-^), hydroxyl radical (·OH), hydrogen peroxide (H_2_O_2_), and organic hydroperoxide (ROOH), which causes oxidative damage to the cell via interactions with cellular components, including lipids, DNA, and proteins [3]. These reactions lead to lipid peroxidation, DNA mutation, DNA-protein crosslinking, protein oxidation, and fragmentation. *P*. *aeruginosa* has evolved mechanisms to protect itself from oxidative stress to survive during these conditions. Several antioxidant enzymes degrade ROS toxicity, such as catalases, superoxide dismutases, alkyl hydroperoxide reductases, and thiol peroxidases [4–6]. Antioxidant molecules, such as vitamins and glutathione (GSH), also play roles in ROS removal. Biomolecular repair enzymes, such as methionine sulfoxide reductases (MSR), are required during high oxidative damage conditions [7].

The tripeptide GSH is a thiol molecule that is found in most Gram-negative bacteria and all eukaryotic cells [8]. GSH is an important compound in cells because it is involved in the maintenance of cellular homeostasis, regulation of sulfur transport, conjugation of metabolites, xenobiotic detoxification, antibiotic resistance, enzymatic regulation, and the expression of stress response genes [9]. GSH is the most abundant antioxidant molecule in cells, and it protects against oxidative stress via direct and indirect interactions with ROS [10]. GSH donates its electrons directly to O_2_·^-^, ·OH, peroxy radical (ROO·), and peroxynitrite (ONOO^-^), which leads to glutathione disulfide (GSSG), and glutathione peroxidase decompose H_2_O_2_ using GSH [3]. GSH reacts with free radicals, and it is oxidized to form GSSG [8]. Glutathione reductase reduces GSSG back to GSH for recycling during the redox process in cells [8].

A two-step process catalyzed by γ-glutamylcysteine synthetase and glutathione synthetase is required to synthesize GSH. γ-Glutamylcysteine synthetase is encoded by the *gshA* gene, and it catalyzes the bonding formation between glutamate and cysteine to form γ-L-glutamylcysteine [8]. Glutathione synthetase is encoded by the *gshB* gene, and it catalyzes the formation of the addition glycine and cysteine in γ-L-glutamylcysteine to form GSH [8]. *Escherichia coli* that lack the GSH biosynthesis gene (*gshA* or *gshB*) are sensitive to diamide [11]. The absence of *gshA* in *Salmonella sp*. increased susceptibility to H_2_O_2_ and nitrosative stress [12]. *Salmonella* without *gshA* exhibited attenuated virulence in a murine model [12].

The aim of this work was to investigate the roles of glutathione biosynthesis genes *gshA* (*PA5203*) and *gshB* (*PA0407*) in the oxidative stress protection and bacterial virulence of *P*. *aeruginosa*.

## Results and discussion

### Expression profiles of *gshA* and *gshB* in response to stress

**Fig 1:**
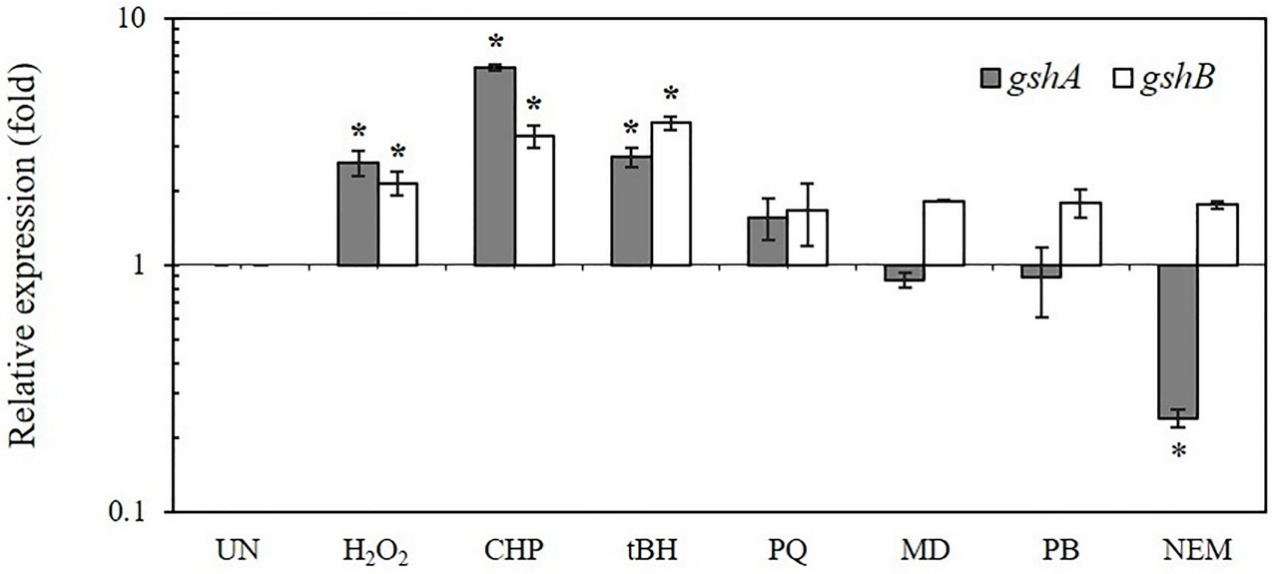
The expression of *gshA* and *gshB* in response to stress. The expression levels of *gshA* and *gshB* were determined using real-time RT-PCR. Exponential-phase cells of *P*. *aeruginosa* PAO1 were subjected to various stress conditions, including 1 mM H_2_O_2_, 1 mM CHP, 1 mM tBH, 0.5 mM PQ, 0.5 mM MD, 0.5 mM PB, and 0.5 mM NEM for 15 min prior to RNA preparation for real-time RT-PCR analysis. Relative expression (RE) was normalized to the *16S rRNA* gene, and results are expressed as the fold-expression relative to the level of uninduced condition (log_10_ RE = 1). Data shown are means ± SD of three independent experiments. The asterisk indicates a statistically significant difference (*p* < 0.05 by one-way ANOVA with Bonferroni correction).

The *P*. *aeruginosa* PAO1 genome contains *gshA* (*PA5203*), which encodes the glutamate-cysteine ligase, and *gshB* (*PA0407*), which encodes γ-glutamylcysteine synthetase and glutathione synthetase. The gene expression patterns of *gshA* and *gshB* under stress conditions were investigated using real-time RT-PCR. PAO1 cultures were challenged with 1 mM H_2_O_2_, superoxide generators (0.5 mM plumbagin [PB], 0.5 mM menadione [MD], and 0.5 mM paraquat [PQ]), organic hydroperoxides (1 mM cumene hydroperoxide [CHP], and 1 mM *t*-butyl hydroperoxide [tBH]) and a thiol-depleting agent (0.5 mM N-Ethylmaleimide [NEM]). Fig 1 shows that peroxides, including H_2_O_2_ (2.6 ± 0.3-fold), CHP (6.3 ± 0.2-fold), and tBH (2.7 ± 0.2-fold), considerably increased *gshA* expression compared to uninduced levels. However, other oxidants, including superoxide generators and NEM, did not significantly induce *gshA* expression (Fig 1). Exposure to H_2_O_2_ (2.1 ± 0.2-fold), CHP (3.3 ± 0.4-fold), and tBH (3.7 ± 0.3-fold) increased gshB expression, but PQ, MD, PB, and NEM treatments only marginally induced expression (approximately 50%) compared to the uninduced condition. There were some similarities between the patterns of *gshA* and *gshB* expression. Notably, treatment of PAO1 with MD and PB induced a small (approximately 40%) reduction in *gshA* expression compared to PAO1. NEM treatment produced an over 4-fold reduction in *gshA* expression (Fig 1). These treatments unexpectedly induced a small increase in *gshB* expression (2-fold) (Fig 1). The contrasting patterns of *gshA* and *gshB* responses to these oxidants suggest a complex response involving GSH and its intermediates. The oxidant expression profiles of *gshA* and *gshB* shared some similarities, but these patterns did not fit any known oxidant sensing/responding transcription regulators (IscR, Fur, or OxyR) [13, 14]. These novel patterns suggest that single or multiple unknown regulators differentially modulated these two genes. These hypotheses are being investigated. The oxidant expression profiles of these genes suggest that these genes play a role in protecting cells from oxidants that highly induce their expression [15, 16].

### *gsh* mutants exhibit increased susceptibilities to paraquat (PQ) and N-Ethylmaleimide (NEM)

**Fig 2:**
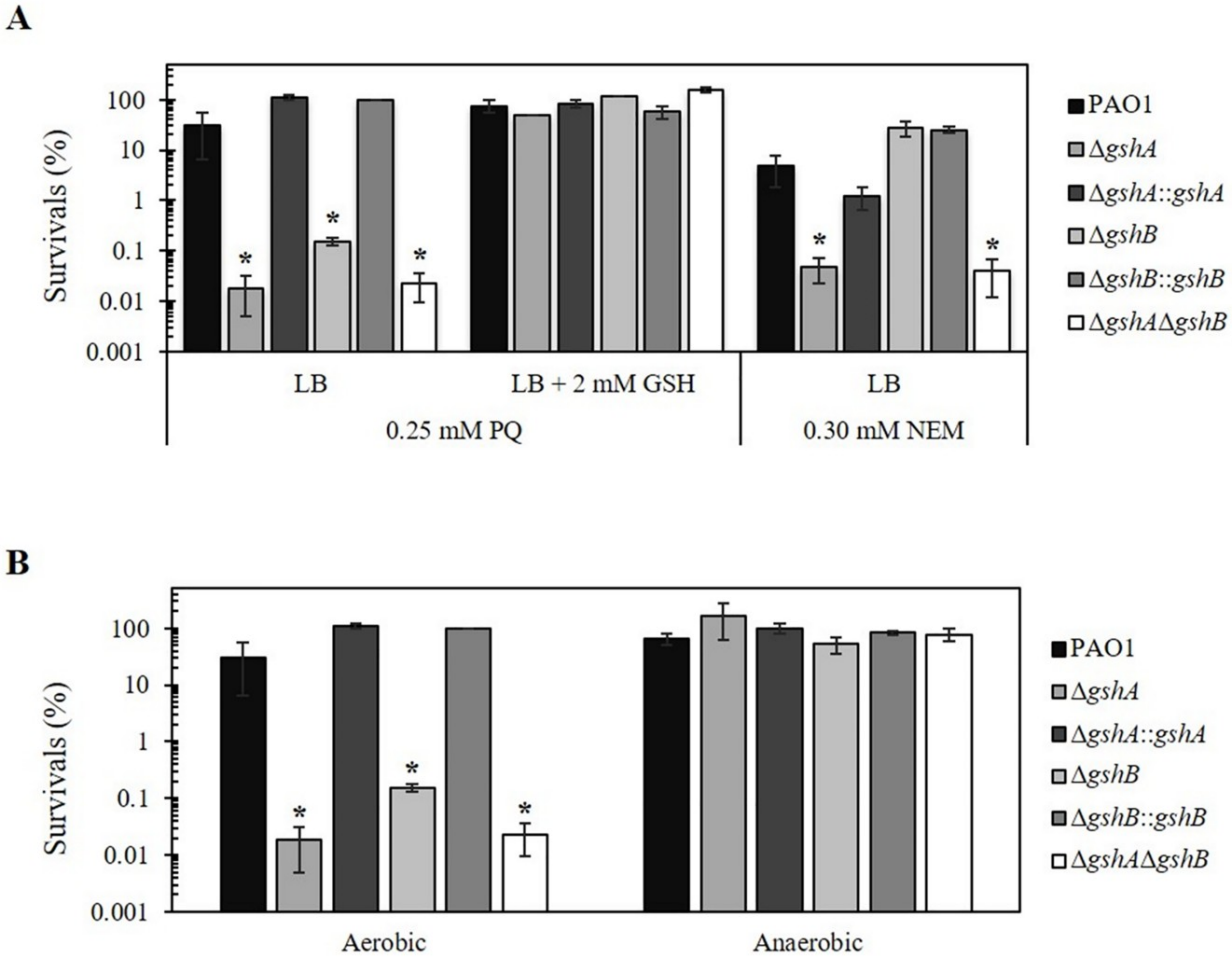
Determination of PQ and NEM resistance levels in *gsh* mutants and PAO1. (A) Plate sensitivity assay was performed with and without 2 mM GSH supplementation in LB plates containing 0.25 mM PQ and 0.30 mM NEM. (B) Plate sensitivity assay against 0.25 mM PQ using LB plates plus 1% NaNO_3_ and incubated under aerobic and anaerobic conditions. Data presented are means ± SD of three independent experiments. The asterisk indicates a statistically significant difference (*p* < 0.01 by one-way ANOVA with Bonferroni correction) relative to PAO1.

A plate sensitivity assay was performed to compare plate growth efficiency in the presence of oxidants between the PAO1 and *gsh* mutants and investigate the physiological roles of GshA and GshB in oxidative stress protection. All *gsh* mutants, including Δ*gshA*, Δ*gshB*, and double Δ*gshA*Δ*gshB* mutants, were 10^3^-fold more sensitive to PQ (0.25 mM) treatment compared to wild-type PAO1 (Fig 2A). The PQ-sensitive phenotype of Δ*gshA* and Δ*gshB* was complemented in the mutant strains transposed with a mini-Tn7 vector containing the full-length gene and showed levels similar to PAO1, which suggests that the PQ susceptibility in these mutants was the result of a lack of functional GshA or GshB. Exogenous GSH (2 mM) was supplemented in the medium to confirm whether GSH, which is a product of GshA and GshB, was involved in protection against PQ toxicity in *P*. *aeruginosa*. Bacterial survival was determined after 0.25 mM PQ treatment. The survival rates of Δ*gshA*, Δ*gshB*, double Δ*gshA*Δ*gshB* mutants, and PAO1 grown in 2 mM GSH supplemented medium after paraquat treatment increased significantly (10^3^-fold) in all *gsh* mutants compared to mutants grown in LB without 2 mM GSH (Fig 2A). There were no significant differences in survival rates after paraquat treatment in PAO1, Δ*gshA*::*gshA*, and *gshB*::*gshB* complemented strains grown in LB with 2 mM GSH compared with strains grown in LB alone (Fig 2A). This result suggests that the PQ susceptibility in these *gsh* mutants resulted from defects in glutathione biosynthesis and the exogenous GSH restored the PQ-sensitive phenotype. This result suggests that GSH plays a role in the protection against PQ toxicity in PAO1. A similar observation has shown that *P*. *aeruginosa gshA* mutant is more sensitive to PQ [17].

PQ is a superoxide generator that undergoes an intracellular redox cycling reaction via the acceptance of an electron from NADPH and transfers that electron to oxygen to produce a superoxide anion [18]. However, PQ itself exerts its toxicity in an oxygen-independent manner via intracellular transformations [19]. The plate sensitivity assay was performed under aerobic and anaerobic conditions, as described in the Materials and Methods, to determine whether PQ toxicity in *gsh* mutants was produced from superoxide anion generation or direct toxicity. The 10^3^-fold increase in PQ sensitivity of Δ*gshA*, Δ*gshB*, and Δ*gshA*Δ*gshB* mutants was abolished under the anaerobic condition compared to the aerobic condition (Fig 2B). These *gsh* mutants exhibited a similar PQ susceptibility under the anaerobic condition as PAO1. Therefore, the increased PQ susceptibility of the Δ*gsh* mutant might require oxygen and most likely resulted from superoxide anion-mediated toxicity. These results support the hypothesis that GSH acts as an antioxidant agent to scavenge this superoxide radical and defects in GSH biosynthesis contribute to oxidative stress that leads to cell death [17].

The Δ*gshA* and double Δ*gshA*Δ*gshB* mutants were 10^2^-fold more susceptible to NEM (0.30 mM) than the wild type (Fig 2A). However, the Δ*gshB* mutant exhibited similar susceptibility levels to NEM as the wild type (Fig 2A), which suggests that the lack of GshB activity did not affect thiol-depletion. Complementation of the *gshA* mutant (Δ*gshA*::*gshA*) strain produced similar susceptibility levels as PAO1 (Fig 2A).

NEM is a thiol-depleting compound that reacts with the sulfhydryl group of cysteine residues in several proteins. NEM causes cellular thiol depletion and contributed to the NEM hypersensitive phenotype of *gshA* mutant, which suggests that GSH biosynthesis is important to NEM resistance. GSH protects proteins from NEM-induced modification to maintain the function of these proteins under NEM exposure [20]. GSH also reacts chemically with NEM to lower toxic concentrations [20]. The deletion of *gshA* resulted in the lack of GSH and its intermediates, and cells with deleted *gshB* gene still produced γ-glutamylcysteine, which is an intermediate of GSH biosynthesis that exhibits antioxidant properties [11]. GSH detoxifies NEM toxicity via direct conjugation to produce an N-ethylsuccinimido-S-glutathione (ESG) adduct [20], which activates potassium efflux systems and decreases cytoplasmic pH to protect cells from electrophile toxicity [21]. The ESG adduct is degraded to a non-toxic metabolite, N-Ethylmaleamic acid, during NEM detoxification prior to release from the cell [20]. NEM also activates *P*. *aeruginosa* glutathione-gated potassium efflux (GGKE), which leads to K^+^ and Ca^2+^ efflux and H^+^ influx, and alters biofilms to result in detachment [22]. GSH and its intermediates may provide general thiol-buffering effects to protect bacteria against the thiol-depleting agent NEM.

### *gsh* mutants are sensitive to methylglyoxal

Reactive electrophilic species (RES) are highly reactive molecules that contain α, β-unsaturated carbonyl or electrophilic groups [23]. RES cause stress to the cell via reactions with nucleophilic macromolecules, including proteins and DNA, and produce irreversible damage and mutation [23]. Methylglyoxal is an RES-generating molecule that is highly toxic to cells. The broth microdilution assay was performed using *gsh* mutants to investigate the role of GSH biosynthesis in the protection from methylglyoxal toxicity. The susceptibility level was expressed as the MIC values of each bacterial strain. The Δ*gshA* and Δ*gshA*Δ*gshB* mutants exhibited a 4-fold reduction in MIC level (0.01%) against methylglyoxal, and the Δ*gshB* exhibited a 2-fold reduction in MIC (0.02%) compared to PAO1 (0.04%) (Table 1). The introduction of *gshA* or *gshB* completely restored the increased susceptibility to methylglyoxal of both mutants to the PAO1 level (0.04%). The reduction in MIC against methylglyoxal in *gsh* mutants suggests that cellular GSH is important in the protection of *P*. *aeruginosa* against methylglyoxal-mediated RES, which was observed in other bacteria [24, 25]. Exogenous GSH was supplemented into the culture medium, and the phenotypes were re-examined to determine whether GSH was required for methylglyoxal resistance in this bacterium. Supplementation of 2 mM GSH increased methylglyoxal resistance in *gsh* mutants to levels similar to the PAO1 level (MIC, 0.04%). These results suggest that the methylglyoxal susceptibility of *gsh* mutants resulted from the malfunction of GSH biosynthesis, which decreased GSH levels in the cell.

**Table 1:** MIC of methylglyoxal for *P*. *aeruginosa* PAO1 and *gsh* mutants.

Strains	MIC of Methylglyoxal (%)	
	No GSH	2 mM GSH
PAO1	0.04	0.04
Δ*gshA*	0.01	0.04
Δ*gshA*::*gshA*	0.04	0.04
Δ*gshB*	0.02	0.04
Δ*gshB*::*gshB*	0.04	0.04
Δ*gshA*Δ*gshB*	0.01	0.04

The data shown are the MICs of three independent experiments with SD equal to 0.

RES are produced as by-product of metabolisms in several bacteria, animals, and human. Methylglyoxal is generated by the fragmentation of triose phosphates (a glycolysis intermediate). Then, dihydroxyacetone phosphate (DHAP, a glycolysis intermediate) could be converted to methylglyoxal by methylglyoxal synthase [23, 26]. Methylglyoxal uses different mechanisms to exert its antimicrobial activity, including inhibition of protein, DNA, and RNA synthesis [27, 28]. Bacterial methylglyoxal detoxification is carried out mainly by glyoxalase I and II enzymes [23]. In *E*. *coli*, glyoxalase I requires GSH as a cofactor in the converting of methylglyoxal to the intermediate S-lactoylglutathione, which activates the potassium efflux pump and NEM-GSH adduct (ESG) [26]. The acidic cytoplasm contributes to cell survival against the methylglyoxal toxicity. Notably, *P*. *aeruginosa* expresses two glyoxalase I enzymes, which belong to different metal activation classes [29]. Glyoxalase II further converts S-D-lactoylglutathione to glycolic and lactic acids [23]. Therefore, cellular GSH plays a direct role in the full activity of glyoxalase I in the detoxification of methylglyoxal in this bacterium.

### Δ*gsh* mutants attenuate virulence in a *Drosophila* host model

**Fig 3:**
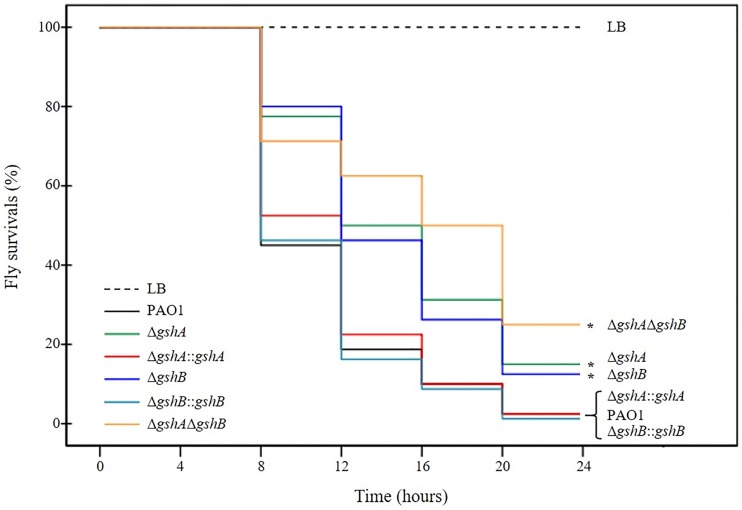
Kaplan-Meier survival curve of fruit fly *Drosophila melanogaster* infected with PAO1 and mutants. Each group of 20 *D*. *melanogaster* was infected with PAO1, *gsh* mutants, and complemented strains. Experiments were independently repeated for six times and the pooled data were used to build the survival curves (n = 120). Negative control groups were treated with fresh LB medium. The asterisk indicates a statistically significant difference. Statistical significance was measured by using Log-Rank test *p* < 0.01 as compared to the wild type.

GSH is responsible for ROS and RES protections in *P*. *aeruginosa*, and these factors contribute to bacterial pathogenicity. The virulence of the *P*. *aeruginosa gsh* mutant strains was tested using a fruit fly *Drosophila melanogaster* feeding assay, as described in the Materials and Methods. The Kaplan-Meier survival curves showed that median lifespans of flies infected with Δ*gshA*, Δ*gshB*, and Δ*gshA*Δ*gshB* mutants were significantly increased to 12, 12, and 16 h, respectively, compared to 8 h in PAO1 (Log-Rank *p* < 0.01) (Fig 3). Loss of *gshA* and *gshB* genes in *P*. *aeruginosa* extended lifespan of the infected *Drosophila*. The Δ*gshA*, Δ*gshB*, and Δ*gshA*Δ*gshB* mutants had reduced virulence compared to the PAO1, as indicated by a greater survival of infected flies. The wild-type PAO1 infected flies showed 10 ± 2.8% fly survival compared to 100% LB feeding as a negative control after a 16 h incubation. The Δ*gshA*, Δ*gshB*, and Δ*gshA*Δ*gshB* mutants showed 31 ± 3%, 26 ± 2.8%, and 50 ± 5.8% fly survival at 16 h, respectively, compare to that of the PAO1. The percentage of fly survival increased significantly at 8–16 h time intervals when the Δ*gshA*, Δ*gshB*, and Δ*gshA*Δ*gshB* mutants were fed to *D*. *melanogaster*, respectively (*p* < 0.05 by one-way ANOVA with Bonferroni correction) relative to PAO1. These results indicate that deletion of *gshA* or *gshB* attenuates the virulence of *P*. *aeruginosa* PAO1 in the tested model. Functional *gshA* or *gshB* restored the attenuated virulence in these mutants because similar levels of percent fly survival as the wild-type flies were observed at the same time (10 ± 1.6% and 8.5 ± 3% fly survival, respectively). These results suggest that GSH biosynthesis plays important roles in the pathogenicity of *P*. *aeruginosa* in the fruit fly *Drosophila* host model.

Fly immunity is a multilayered system that includes at least 7 defense mechanisms to protect flies from invading pathogens [30]. One of these mechanisms that regulates bacteria in the fly gut is antimicrobial peptides (AMPs), and ROS, particularly superoxide anions produced from midgut epithelial cells, is a first-line defense mechanisms [31]. In the infected fly gut, ROS was produced from the NADPH oxidase (DUOX) protein of epithelial cells, which is triggered by invading bacteria [32]. *P*. *aeruginosa* PAO1 protected itself from the oxidative stress generated by the host cells via the use of GSH as an antioxidant agent, which lead to growth in the fly gut and host death from bacterial infection. GSH is responsible for virulence attenuation and the superoxide hypersensitivity of *gsh* mutants. Loss of *gshA* or *gshB* in *P*. *aeruginosa* attenuated the virulence ability to cause fly death, likely because of a reduced ability to survive within the host. Therefore, *gsh* mutants were killed more rapidly by host-produced ROS.

Virulence factors play an important role in bacterial infection, colonization, and invasion within the host cell [33]. Different virulence factors are required in two forms of bacterial stages during infection: the planktonic form is involved in acute infection, and biofilm is involved in chronic infection [34]. Planktonic bacteria produce several virulence factors to infect the host, including phenazine pyocyanin, which generates ROS and promotes inflammation, motility factors that facilitate bacterial movement through host cells, siderophores that trap extracellular iron, and toxins that damage host cells [34]. Biofilm formation is associated with persistent infection and antibiotic resistance within host cells [35].

### Glutathione plays important roles in pyocyanin production

**Fig 4:**
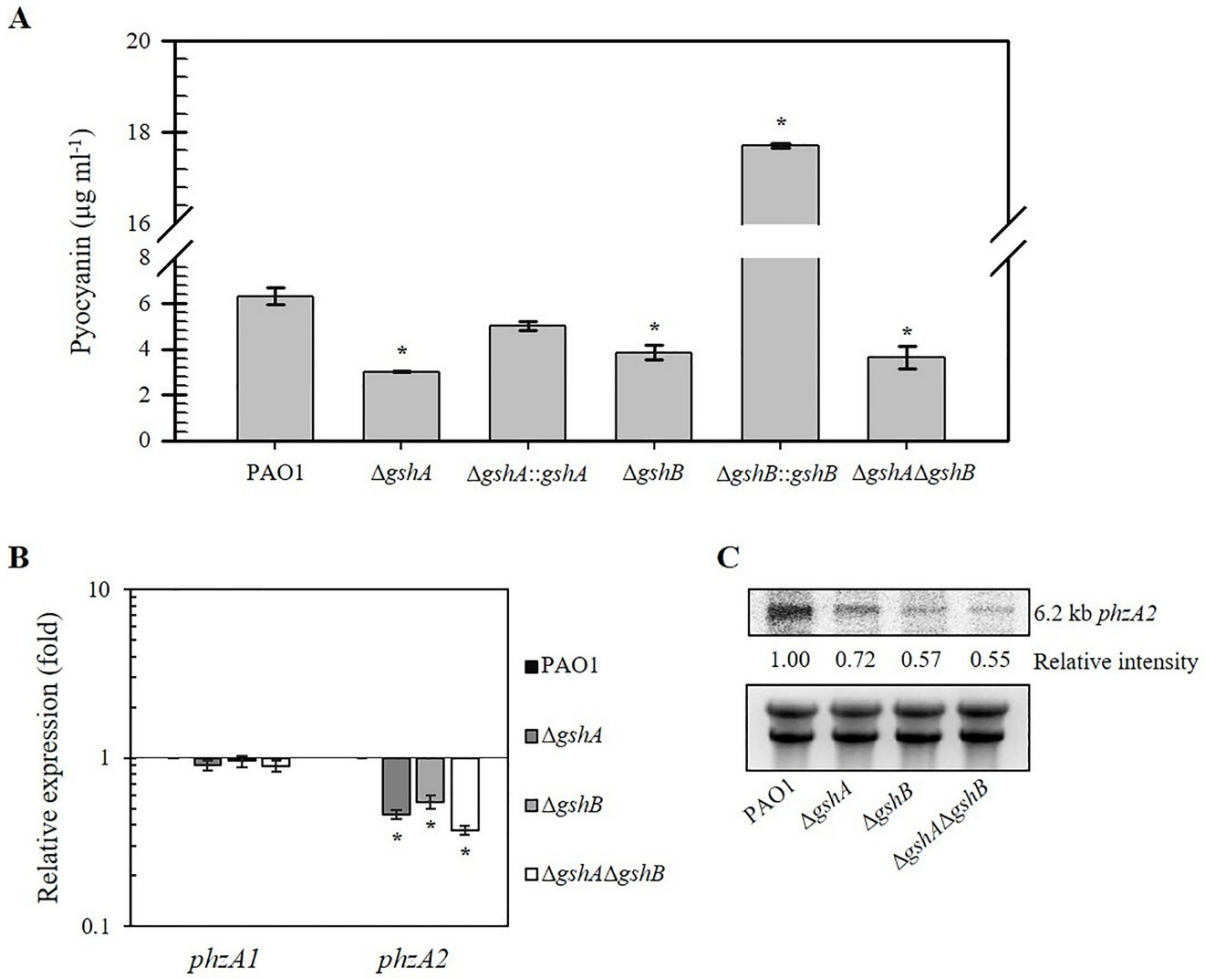
Pyocyanin production in *P*. *aeruginosa* wild-type PAO1 and *gsh* mutants. (A) PAO1, Δ*gshA*, Δ*gshA*::*gshA*, Δ*gshB*, Δ*gshB*::*gshB* and double Δ*gshA*Δ*gshB* mutant strains were cultured for 24 h, and the supernatant was collected for pyocyanin measurement. (B) Real-time RT-PCR analysis of *phzA1* and *phzA2* expression. Total RNA was isolated from PAO1, Δ*gshA*, Δ*gshB*, and double Δ*gshA*Δ*gshB* strains. Data shown are the fold change in expression relative to wild-type PAO1 level. (C) Northern blot analysis of mRNA samples probed with radioactively labeled *phzA2*. Total RNA (20 μg) prepared from the cultures of PAO1 and the *gsh* mutants were loaded into each lane. The number below each band represents the fold change in band intensity relative to the level of the wild type determined using densitometric analysis. The asterisk indicates a statistically significant difference (*p* < 0.01 by one-way ANOVA with Bonferroni correction) compared with PAO1.

*P*. *aeruginosa* pyocyanin is a terminal signaling factor in a quorum sensing network and a virulence factor from oxidative stress pathways, which is involved in the pathophysiological effects in cystic fibrosis patients [36]. The amount of pyocyanin in *gsh* mutant strains was measured and compared to PAO1. The results in Fig 4A show that the wild-type PAO1 culture medium contained 6.32 ± 0.3 μg ml^-1^ pyocyanin, and the Δ*gshA*, Δ*gshB*, and double Δ*gshA*Δ*gshB* mutant culture medium contained significantly lower pyocyanin (3.01 ± 0.0, 3.84 ± 0.3, and 3.63 ± 0.4 μg ml^-1^, respectively). The amount of pyocyanin in the culture medium of the Δ*gshA*::*gshA* complemented strains was similar to PAO1, and the Δ*gshB*::*gshB* strain exhibited increased pyocyanin levels up to 17.72 ± 0.05 μg ml^-1^ (3-fold higher than PAO1). The effect of constitutive expression of *gshB* from the Tn7 expression vector promoter on pyocyanin production was unexpected. This result may be due to deregulation of *gshB* expression, which leads to a much higher level of pyocyanin via unknown mechanisms. The significantly decreased pyocyanin levels in these *gsh* mutants suggest that bacterial GSH biosynthesis is required for pyocyanin production. *P*. *aeruginosa gshA* mutants have been shown to produce reduced pyocyanin [17].

Production of pyocyanin in *P*. *aeruginosa* involves two homologous systems encoded by the *phzA1B1C1D1E1F1G1* (*phzA1*) and *phzA2B2C2D2E2F2G2* (*phzA2*) gene clusters [37]. The expression of *phzA1* and *phzA2* in *gsh* mutants was determined using real-time RT-PCR. The level of *phzA1* exhibited a small decrease (less than 50%) compared to PAO1 levels in *gsh* mutants (Fig 4B). The expression of *phzA2* was 2-fold lower in the Δ*gshA*, Δ*gshB*, and double Δ*gshA*Δ*gshB* mutants compared with the PAO1 (Fig 4B). These results suggest that GSH biosynthesis is required for the full expression of *phzA1* and *phzA2* operons via unknown mechanisms, and the expression levels of these operons contribute to overall pyocyanin production in *P*. *aeruginosa* [38]. Northern blot analysis was performed to confirm the expression of *phzA* in the *gsh* mutants compared to PAO1. The results demonstrate that *phzA2* genes were transcribed mostly as polycistronic transcripts, and *phzA*2 expression was reduced approximately 2-fold in the Δ*gshA*, Δ*gshB*, and double Δ*gshA*Δ*gshB* mutants relative to PAO1 level (Fig 4C). These results are consistent with the results of the real-time RT-PCR analysis. A similar observation was reported for the *gshB* mutant in *P*. *aeruginosa* [38].

GSH interferes with the ability of pyocyanin to interact with extracellular DNA (eDNA) via a direct reaction with pyocyanin [39]. The reaction of eDNA and pyocyanin is important in biofilm formation. Therefore, the balance of pyocyanin-eDNA-GSH was altered in the absence of GSH, which could lead to the observed decrease in pyocyanin production (Fig 4A).

### *gsh* mutants produce lower pyoverdine levels

**Fig 5:**
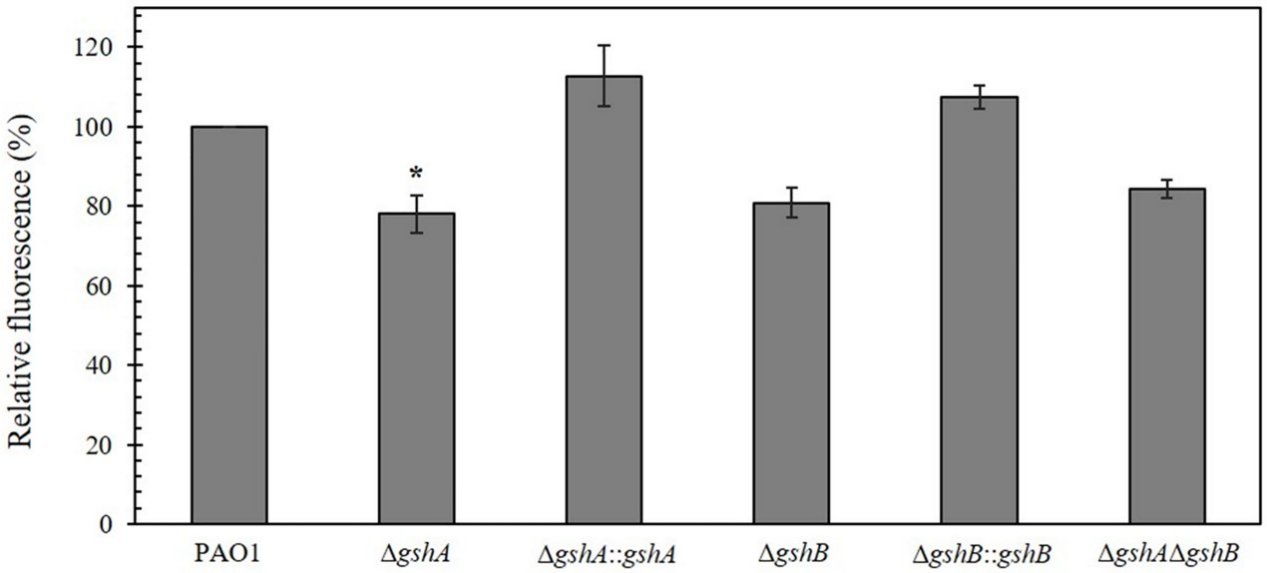
Pyoverdine production in *P*. *aeruginosa*. PAO1, Δ*gshA*, Δ*gshA*::*gshA*, Δ*gshB*, Δ*gshB*::*gshB* and double Δ*gshA*Δ*gshB* mutants were incubated in *Pseudomonas* F medium overnight at 37 °C. Pyoverdine was fluorometrically measured by recording the emission at 477 nm and excitation at 400 nm in a luminescence spectrometer. The asterisk indicates a statistically significant difference (*p* < 0.05 by one-way ANOVA with Bonferroni correction) compared to PAO1.

Pyoverdine is a green fluorescent siderophore that is also involved in *P*. *aeruginosa* pathogenicity. It is secreted from *P*. *aeruginosa* under iron-limiting conditions for the chelation of ferric ions in the environment into cells [40]. It has been shown that pyoverdine is essential for *P*. *aeruginosa* virulence [41]. Addition of GSH to clinically isolated *P*. *aeruginosa* strain has been shown to increase pyoverdine concentration in both the pyoverdine and ferripyoverdine forms [42]. The amount of pyoverdine in PAO1 and *gsh* mutants were quantified using fluorescent spectrometry. The intensity of fluorescence was normalized to cell density (OD_600nm_), and the results are shown as percent relative fluorescence intensity. The percent relative fluorescence intensity of pyoverdine in Δ*gshA*, Δ*gshB*, and double Δ*gshA*Δ*gshB* mutants were 78.0 ± 2.4%, 80.9 ± 1.8%, 84.3 ± 1.1%, respectively, compared with 100% for PAO1 (Fig 5). Hence, the amount of pyoverdine produced by *gsh* mutants were 15 to 20% lower than PAO1. The expression of functional *gshA* and *gshB* restored pyoverdine secretion in the GSH mutants to the PAO1 level (Fig 5). These data suggest that GSH biosynthesis is involved in pyoverdine production in *P*. *aeruginosa*.

Siderophore pyoverdine is secreted by the type VI secretion system (T6SS) under iron-limiting conditions in *P*. *aeruginosa* to scavenge Fe^3+^ in the extracellular environment [43]. Ferripyoverdine (pyoverdine-Fe^3+^ complex) binds the FpvA outer membrane receptor and imports iron into the cell. FpvA interacts with FpvR antisigma factor in the periplasm and transmits the signal to the cytoplasmic domain of FpvR. Two sigma factors, σ^PvdS^ and σ^FpvI^, are activated and bind to RNA polymerase to initiate the transcription of pyoverdine synthesis genes and *fpvA*, respectively [40].

Another siderophore in *P*. *aeruginosa* is pyochelin. Ferripyochelin is transported into the cell via the FptA outer membrane receptor [44]. Ferripyochelin iron reductase located in the periplasm and cytoplasm catalyzes the reduction of Fe^3+^ and releases Fe^2+^ from the pyochelin [44]. GSH and NADH are electron donators for ferripyochelin iron reductase [44]. Therefore, the re-adjustment of iron uptake processes, such as reduced pyoverdine synthesis, is required in the absence of thiols.

### Δ*gsh* mutants exhibit impaired swimming and twitching motility

Motility is an essential factor contributing to the bacteria virulence [45]. Flagella and type IV pili play important roles in bacterial virulence during acute and chronic *P*. *aeruginosa* infections. Flagella are also required for biofilm formation and contribute to persistent colonization, and type IV pili mediate adherence to the epithelial cell surface and contribute to biofilm formation [46]. *P*. *aeruginosa* uses a single flagellum for swimming in a liquid environment, and twitching is flagellum-independent. Twitching is powered by an extension and retraction of pili. Flagellar assembly is required the correct folding proteins in either motor switch or hook for efficient movement. GSH is involved in disulfide bond formation and protein folding [47]. This study, we assessed whether GSH deficiency affect bacterial cell motility. Table 2 shows that the swimming motility of wild-type PAO1 was 47.0 ± 1.7 mm after 48 h of incubation. Defects in swimming motility in the Δ*gshA* (33.0 ± 2.0 mm), Δ*gshB* (38.5 ± 2.1 mm), and Δ*gshA*Δ*gshB* mutants (35.0 ± 2.0 mm) were observed compared to PAO1. The Δ*gshA*::*gshA* and the Δ*gshB*::*gshB* complemented strains exhibited restored motility (43.3 ± 1.2 and 45.3 ± 5.7 mm, respectively) to a similar level as PAO1 (Table 2). Table 2 also shows that twitching motility was reduced significantly in the Δ*gshA*, Δ*gshB*, and Δ*gshA*Δ*gshB* mutants (32.3 ± 3.1, 33.7 ± 3.2, and 32.3 ± 2.5 mm, respectively) compared to PAO1 (43.3 ± 1.5 mm). Swimming and twitching motility were defective in the Δ*gsh* mutants, but the colony sizes of these mutants on the LB agar were similar to PAO1 (data not shown).

**Table 2:** Swimming and twitching motility in *P*. *aeruginosa* after 48 h incubation.

Strains	Motility Zone (mm ± SD)	
	Swimming	Twiching
PAO1	47.0 ± 1.7	43.3 ± 1.5
Δ*gshA*	33.0 ± 2.0 *	32.3 ± 3.1 *
Δ*gshA*::*gshA*	43.3 ± 1.2	43.0 ± 2.0
Δ*gshB*	38.5 ± 2.1 *	33.7 ± 3.2 *
Δ*gshB*::*gshB*	45.3 ± 5.7	41.3 ± 1.5
Δ*gshA*Δ*gshB*	35.0 ± 2.0 *	32.3 ± 2.5 *

The data shown are the means ± SD of motility zone (mm) at 48 h incubation of three independent experiments. The asterisk indicates a statistically significant difference (*p* < 0.01 by one-way ANOVA with Bonferroni correction) relative to PAO1.

GSH is transported from the bacterial cytoplasm to the periplasm via the CydDC transporter, which is an ATP-binding cassette-type transporter [48]. GSH exportation maintains the redox environment and protects cells from external toxicity or electrophilic compounds in *S*. *typhimurium* and *E*. *coli* [49]. Periplasmic GSH participates in disulfide bond formation and protein folding via the disulfide bond protein (Dsb) pathway [47]. These correct folding proteins are important for the proper assembly of flagellar motors and pili. *E*. *coli cydD* mutants exhibited defective cell motility due to disrupted flagellar assembly. The *gshA* mutant also exhibited defective flagella function. These results indicate that decreased GSH level could affect cell motility [17].

### Deletion of genes encoding glutathione biosynthesis increases biofilm formation

**Fig 6:**
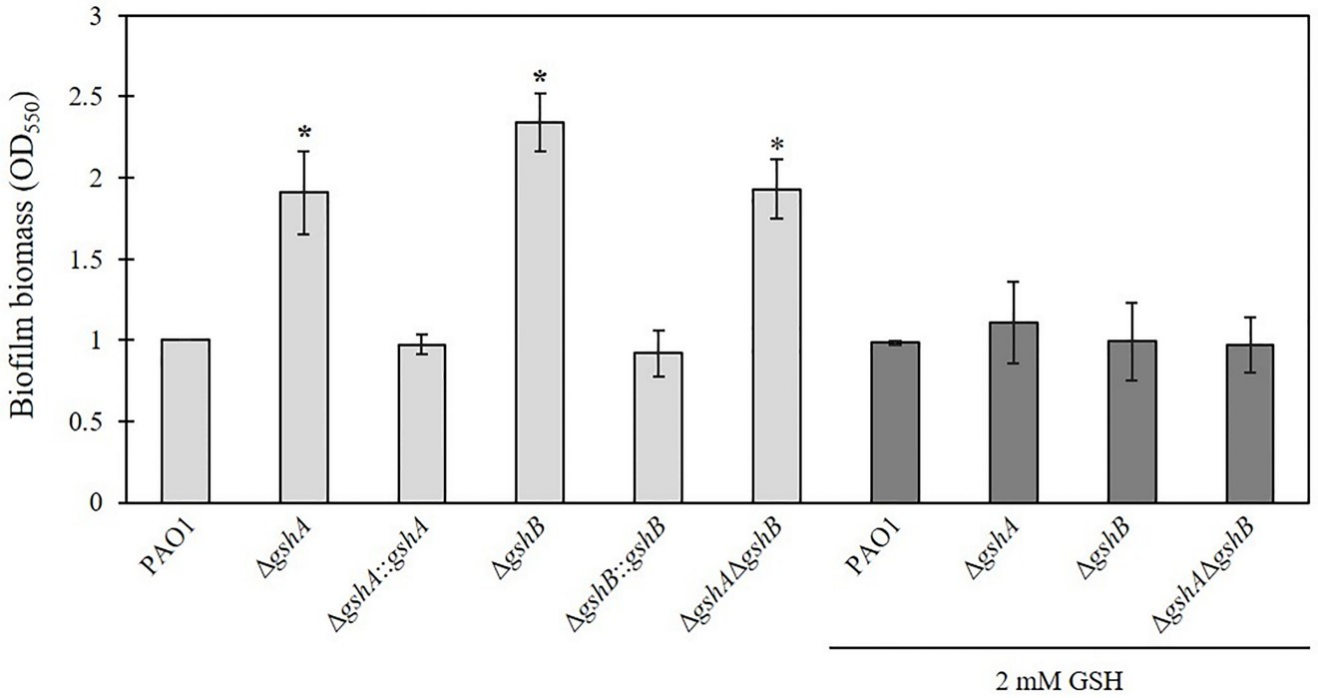
Biofilm formation assay in *P*. *aeruginosa*. PAO1, Δ*gshA*, Δ*gshA*::*gshA*, Δ*gshB*, Δ*gshB*::*gshB* and double Δ*gshA*Δ*gshB* mutants were cultured in LB medium with or without 2 mM GSH supplementation for 24 h. The biofilm layer of PAO1 and indicated strains was stained with 1% crystal violet solution. The biofilm biomass (OD_550nm_) of *gsh* mutants and complemented strains compared with PAO1 were quantified. The asterisk indicates a statistically significant difference (*p* < 0.01 by one-way ANOVA with Bonferroni correction) compared to PAO1.

Biofilm formation is one virulence factor in *P*. *aeruginosa*. It is an indicator for the pathogenicity of *P*. *aeruginosa* because it contributes to resistance to various stresses, including antimicrobial stress, and it is an important component of chronic infections [50, 51]. GSH addition disrupted mature and immature biofilms of a clinical *P*. *aeruginosa* strain [42]. Biofilm formation was determined in PAO1 and *gsh* mutants in the present study to assess whether lack of GSH has any effects on biofilm. Biofilm levels in the Δ*gshA*, Δ*gshB*, and double Δ*gshA*Δ*gshB* mutants (1.9 ± 0.2-fold, 2.3 ± 0.1-fold, and 1.9 ± 0.1-fold, respectively) were significantly higher than PAO1 (Fig 6). The Δ*gshA*::*gshA* and Δ*gshB*::*gshB* complemented strains exhibited restored biofilm formation to the PAO1 level (Fig 6). These results demonstrated that a defect in GSH biosynthesis via deletion of *gshA* or *gshB* increased biofilm formation, which suggests a role for GSH in the repression of biofilm formation. Exogenous GSH (2 mM) was added to the bacterial culture during the assay to determine whether GSH inhibited biofilm production in *P*. *aeruginosa*. Fig 6 shows that the addition of GSH decreased biofilm formation in Δ*gshA*, Δ*gshB*, and double Δ*gshA*Δ*gshB* mutants to a level similar to PAO1. These results are contrasted to a previous observation that the *gshA* mutant produced less biofilm [17]. GSH inhibition of biofilm formation in *P*. *aeruginosa* PAO1 is consistent with previous observations that GSH disrupted mature and immature biofilms of a clinical *P*. *aeruginosa* strain [42]. Pyocyanin pigment activates eDNA release from *P*. *aeruginosa* via H_2_O_2_-mediated cell lysis, which leads to the binding of pyocyanin to eDNA and facilitation of biofilm formation [39]. GSH non-enzymatically interacts with pyocyanin to form a pyocyanin-GSH complex that inhibits pyocyanin-mediated cell lysis, the release of eDNA, binding to eDNA, and biofilm formation [39]. A small increase in *gshA* expression levels (1.1-fold) in non-clonal cystic fibrosis isolates *P*. *aeruginosa* was observed when the isolates switched from planktonic to biofilm growth, which suggests that biofilm cells require GSH to control production [52].

GSH biosynthesis was required for the activation of virulence factors in planktonic cells, including pyocyanin pigment, siderophore, and motility to promote virulence, in *P*. *aeruginosa* for infection and survival within the host cell (Figs 4 and 5 and Table 2). GSH acted as an anti-biofilm in chronic infection and adjusted the metabolic protection and stress response mechanisms.

## Conclusion

**Fig 7:**
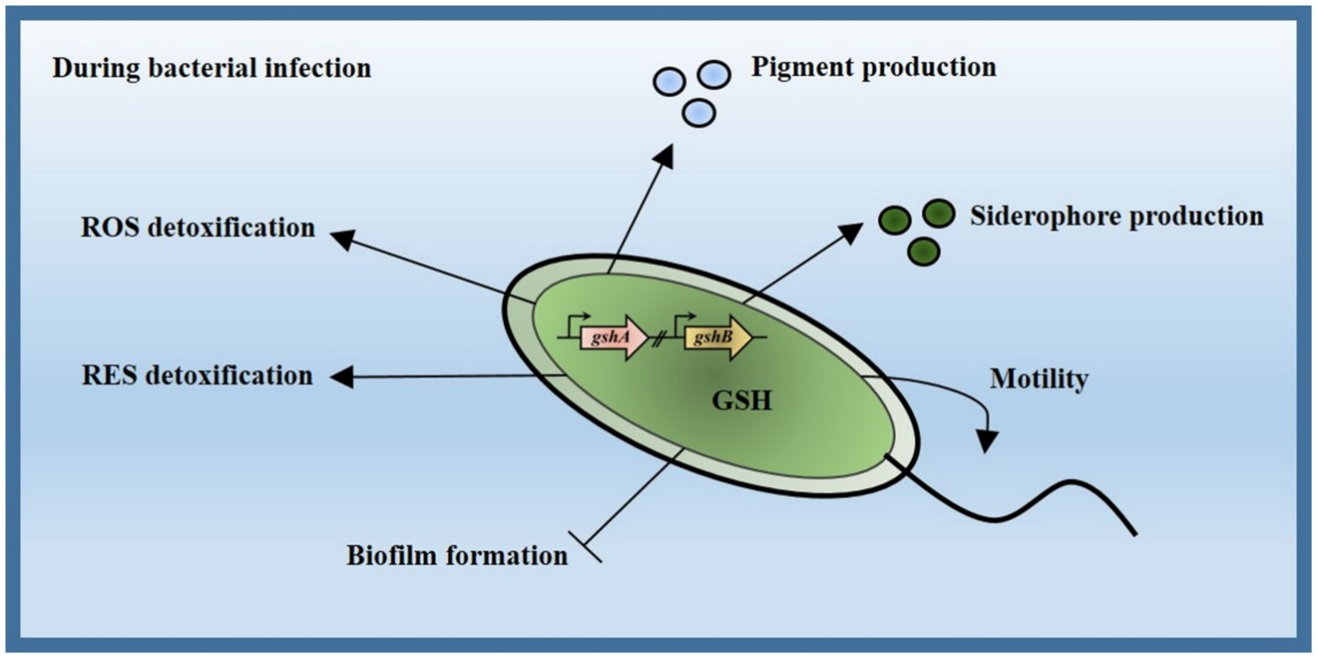
Overview function of GSH biosynthesis in *P*. *aeruginosa*. GshA and GshB catalyzed GSH biosynthesis. Bacteria at the early stage of infection encounter various stresses, including ROS which are generated by the host cell and RES generated from metabolism. GSH biosynthesis plays a primary protective role in the detoxification of these oxidative stresses. *P*. *aeruginosa* required GSH for activation of pigment, siderophore, and motility, which promote bacterial virulence in planktonic cells. GSH also disrupts biofilm formation to control the amount of biofilm.

*gshA* and *gshB* are responsible for GSH biosynthesis in *P*. *aeruginosa*. These findings demonstrated that the inactivation of *gshA* and *gshB* genes increased the susceptibility to ROS- and RES-mediated agents and attenuated virulence due to defects in pigment production, siderophore, and motility (Fig 7). GSH biosynthesis controlled biofilm formation. The data demonstrated that GSH was not essential but played centrally important roles in various physiological processes that were important to survival in the diverse environmental conditions that *P*. *aeruginosa* encountered.

## Materials and methods

### Bacterial strains

All bacterial strains used in this study are listed in S1 Table. *P*. *aeruginosa* strains were grown in Luria-Bertani (LB) with shaking at 180 rpm at 37 °C. The overnight culture was inoculated in fresh LB medium and incubated with shaking. Cells in the exponential phase (optical density at 600 nm [OD_600nm_] of 0.5 after 3 h of growth) were used in all experiments.

### Molecular techniques

General molecular techniques, including DNA and RNA preparations, DNA cloning, PCR amplification, Southern blot analysis, and bacterial transformation, were performed according to standard protocols [53].

### Expression analysis of *gshA*, *gshB*, *phzA1* and *phzA2* using real-time RT-PCR

Total RNA samples were extracted from exponential phase PAO1 culture (OD_600nm_ of approximately 0.5 after 3 h of growth) with and without oxidant treatment for 15 min at 37 °C. Total RNA was treated with DNase I (Thermo Scientific, USA) prior to performing cDNA synthesis using 5 μg DNase I-treated RNA, RevertAid Reverse Transcriptase, and random hexamer primers (Thermo Scientific, USA). Real-time PCR was performed as previously described [54] using 10 ng cDNA to determine gene expression levels using primers EBI11 (5’-CGCTACGGCAAGACCATG-3’) and EBI12 (5’-GCGCTCCAACTGGCTCGG-3’) for *gshA*, BT5458 (5’-CGCATGCGCCCGCTGAAGG-3’) and BT5459 (5’-GCGCGCCAGGCAGTAGGG-3’) for *gshB*, EBI315 (5’-CGGTCAGCGGTACAGGGAA-3’) and EBI316 (5’-GCGAGAGTACCAACGGTTGAAA-3’) for *phzA1*, and EBI316 and EBI317 (5’-CGTCGCACTCGACCCAGAA-3’) for *phzA2*. The *16S rRNA* gene was amplified using primers BT2781 (5′-GCCCGCACAAGCGGTGGAG-3′), and BT2782 (5′-ACGTCATCCCCACCTTCCT-3′) was used as an internal control to normalize cDNA samples. The real-time RT-PCR was performed on Applied Biosystems StepOnePlus. Relative expression analysis was calculated using StepOne software v2.1 and is expressed as fold-expression relative to the level of wild-type PAO1 grown under uninduced condition.

### Northern blot analysis

Total RNA isolation, agarose-formaldehyde gel electrophoresis, blotting, and hybridization were performed as previously described [7, 55]. Purified total RNA (20 μg) was loaded into the gel. A 305-bp fragment of the *phzA2* coding region was used as the DNA probe and amplified using primers EBI316 (5′-GCGAGAGTACCAACGGTTGAAA-3′) and EBI317 (5′-CGTCGCACTCGACCCAGAA-3′). Radioactively labeled probes were prepared using random-primed labeling with [α-^32^P] dCTP.

### Determination of the oxidant resistance level

To determine the oxidant resistance level, a plate sensitivity assay was performed as previously described [56]. Briefly, exponential-phase cells (OD_600nm_ of approximately 0.5 after 3 h of growth) were adjusted to an OD_600nm_ of 0.1 prior to a 10-fold serial dilution in LB medium. Each dilution (10 μl) was spotted onto an LB plate containing 0.25 mM PQ and 0.30 mM NEM. The plates were incubated overnight at 37 °C prior to quantification of colony forming units (CFUs). Percent survival was defined as the CFUs on the plates containing oxidant divided by the CFUs on plates without oxidant and multiplied by 100.

Plate sensitivity assays for anaerobic conditions were performed using LB medium supplemented with sodium nitrate (NaNO_3_, 1% w/v). The culture plates were incubated in an anaerobic jar containing an anaerobic gas pack (AnaeroGen, Oxoid, UK) and incubated at 37 °C for 48 h.

### MIC determination

The minimum inhibitory concentrations (MICs) of methylglyoxal were determined as previously described using a broth microdilution assay [57]. *P*. *aeruginosa* PAO1 and *gsh* mutant strains were grown in LB medium at 37 °C under aerobic conditions until reaching the exponential phase (OD_600nm_ of 0.5). The culture was adjusted to 0.5 McFarland (OD_600nm_ of approximate 0.132) and was diluted 1:20 in fresh medium. The LB broth supplemented with different concentrations of agents with and without 2 mM GSH (Sigma Aldrich, USA) was incubated with bacteria at 37 °C for 18 h. The lowest concentration of antibiotic that inhibited bacterial growth after 18 h of incubation was determined as the MIC value.

### *Drosophila melanogaster* virulence assay

The virulence of *P*. *aeruginosa* and mutants were evaluated using the *Drosophila melanogaster* model as previously described [7, 58]. Essentially, the late-exponential-phase cultures (OD_600nm_ of 0.8) of *P*. *aeruginosa* strains were adjusted to an OD_600nm_ of 0.5 in 800 μl of LB broth prior to the overlaying of cell suspensions onto the surface of preservative-free corn flour *Drosophila* medium (350 ml water, 32 g corn flour, 9 g yeast, 20 g sugar, and 8 g agar) at the bottom of glass fly culture vials. The 12-day-old adult flies were starved for 3 h prior to placement in each vial covered with bacterial cells (20 flies per vial). The vials were incubated at 25 °C, and the number of viable flies was counted every four hours after flies were infected with bacteria.

### Biofilm formation assay

Biofilm formation assays were performed as previously described [59]. Overnight cultures of *P*. *aeruginosa* strains were diluted 1:100 in fresh LB medium, and 150 μl of medium was transferred into 96-well plates. Plates were incubated at 37 °C for 24 h without shaking, and the cell suspension was removed and rinsed with 200 μl of phosphate buffered saline (PBS). The biofilm layer was stained with a 0.1% crystal violet solution and incubated at room temperature for 15 min. The plate was rinsed with water and dried. Ethanol (200 μl) was added to solubilize the crystal violet dye. The absorbance was measured at OD_550nm_ using a spectrophotometer to quantify biofilm formation.

### Bacterial motility assay

Swimming motility was tested in M8 minimal medium supplemented with 1 mM MgSO_4_, 0.5% Casamino acids, 0.2% glucose, and solidified with 0.3% agar for several hours [60]. Overnight bacterial cultures were spotted on agar and incubated at 37 °C for 48 h. Twitching motility was tested using stab inoculation with a toothpick through a 1% agar LB layer to the bottom of the Petri dish and incubated 37 °C for 48 h.

### Pyocyanin pigment production

*P*. *aeruginosa* strains were grown in glycerol alanine minimal medium (GA medium) containing 1% glycerol, 67.3 mM L-alanine, 8 mM MgSO_4_, 0.44 mM K_2_HPO_4_, and 0.065 mM FeSO_4_ at 37 °C for 24 h, and pyocyanin production was quantified based on the absorbance of pyocyanin at 520 nm (OD_520nm_) in an acidic solution as describe previously [61]. Briefly, the supernatant from bacterial culture was collected by centrifugation at 6,000 rpm for 10 min. The pyocyanin in the supernatant was extracted by mixing 3 ml of chloroform into 5 ml of supernatant. The lower chloroform layer containing pyocyanin was collected, and 1 ml of 0.2 M HCl was added to extract pyocyanin into the aqueous phase. The pyocyanin was quantified as OD_520nm_. The pyocyanin concentration is expressed as micrograms of pyocyanin per milliliter of culture supernatant and determined by multiplication of the OD_520nm_ by 17.072 [61].

### Pyoverdine measurement

*P*. *aeruginosa* PAO1 and *gsh* mutant strains were incubated at 37 °C in *Pseudomonas* F medium (BD Difco, USA) overnight. Pyoverdine in the supernatant was fluorometrically measured via recording of the emission at 477 nm and excitation at 400 nm in a luminescence spectrometer and normalized to the OD_600nm_ of the corresponding cultures.

### Statistical analysis

Group data are presented as means ± standard deviation of at least three independent experiments. The level of statistically significant difference between samples was determined using one-way ANOVA with Bonferroni correction, and *p* value of < 0.05 was considered statistically significant.

### Ethics statement

All *P*. *aeruginosa* and *D*. *melanogaster* were raised, maintained and all experiments were conducted following procedures, MUSC2017-001 and MUSC60-052-402, approved by the Committee of Biosafety, Faculty of Science, Mahidol University (MUSC) and the MUSC-Institutional Animal Care and Use Committee (IACUC), respectively.

## Supporting information

S1 Table Bacterial strains used in this study. (DOCX)
