# Supplementary material for: Pseudomonas aeruginosa glutathione biosynthesis genes play multiple roles in stress protection, bacterial virulence and biofilm formation
Source: PLoS One. 2018 Oct 16;13(10):e0205815. doi: 10.1371/journal.pone.0205815 (PMC6191110; doi:10.1371/journal.pone.0205815)
Supplement: S1 Table — (DOCX) [file pone.0205815.s001.docx]

**S1 Table. Bacterial strains used in this study.**

| **Strains** | **Relevant characteristics** | **Source or Reference** |
| --- | --- | --- |
| PAO1 | Wild type carrying mini-Tn7T | Laboratory stock |
| ∆*gshA* | *gshA* mutant, derivative of PAO1 in which a part of *gshA* was deleted and carrying mini-Tn7T | Laboratory stock |
| ∆*gshA*::*gshA* | *gshA* mutant carrying mini-Tn7T containing *gshA* | Laboratory stock |
| ∆*gshB* | *gshB* mutant, derivative of PAO1 in which a part of *gshB* was deleted and carrying mini-Tn7T | Laboratory stock |
| ∆*gshB*::*gshB* | *gshB* mutant carrying mini-Tn7T containing *gshB* | Laboratory stock |
| ∆*gshA*∆*gshB* | Double *gshA* and *gshB* mutant, derivative of *gshA* mutant in which a part of *gshB* was deleted and carrying mini-Tn7T | Laboratory stock |
